# {Bis[2-(diphenyl­phosphan­yl)eth­yl]phenyl­phosphane-κ^3^ *P*,*P*′,*P*′′}[(*Z*)-8-mesityl­cyclo­oct-4-en-1-yl]platinum(II) tetra­fluorido­borate dichloro­methane disolvate

**DOI:** 10.1107/S1600536811023853

**Published:** 2011-06-25

**Authors:** Shu-Bin Zhao, Rui-Yao Wang, Michel R. Gagné

**Affiliations:** a Department of Chemistry, University of North Carolina at Chapel Hill, Chapel Hill, North Carolina 27599, USA; b Department of Chemistry, Queens University, Kingston, Ontario K7L 3N6, Canada

## Abstract

In the title ionic compound, [Pt(C_17_H_23_)(C_34_H_33_P_3_)](BF_4_)·2CH_2_Cl_2_, the Pt^II^ atom adopts a square-planar coordination geometry with the large (*Z*)-8-mesityl­cyclo­oct-4-en-1-yl group occupying the fourth coordination site. The (triphos)Pt moiety and the mesityl group are attached to the cyclo­oct-4-ene motif at the 1- and 8-position in a *syn* configuration. The (BF_4_)^−^ anion and one of the dichloromethane solvate molecules each are disordered over two sets of sites.

## Related literature

For structures of similar triphos-chelating Pt^II^-alkyl compounds, see: Koh & Gagné (2004 ▶); Feducia & Gagné (2008 ▶); Sokol *et al.* (2011 ▶). For structures of compounds incoporating cyclooctadiene (COD) and also generated *via* coordination-triggered bond metal-carbon migratory insertion reactions, see: Lin *et al.* (2009 ▶).
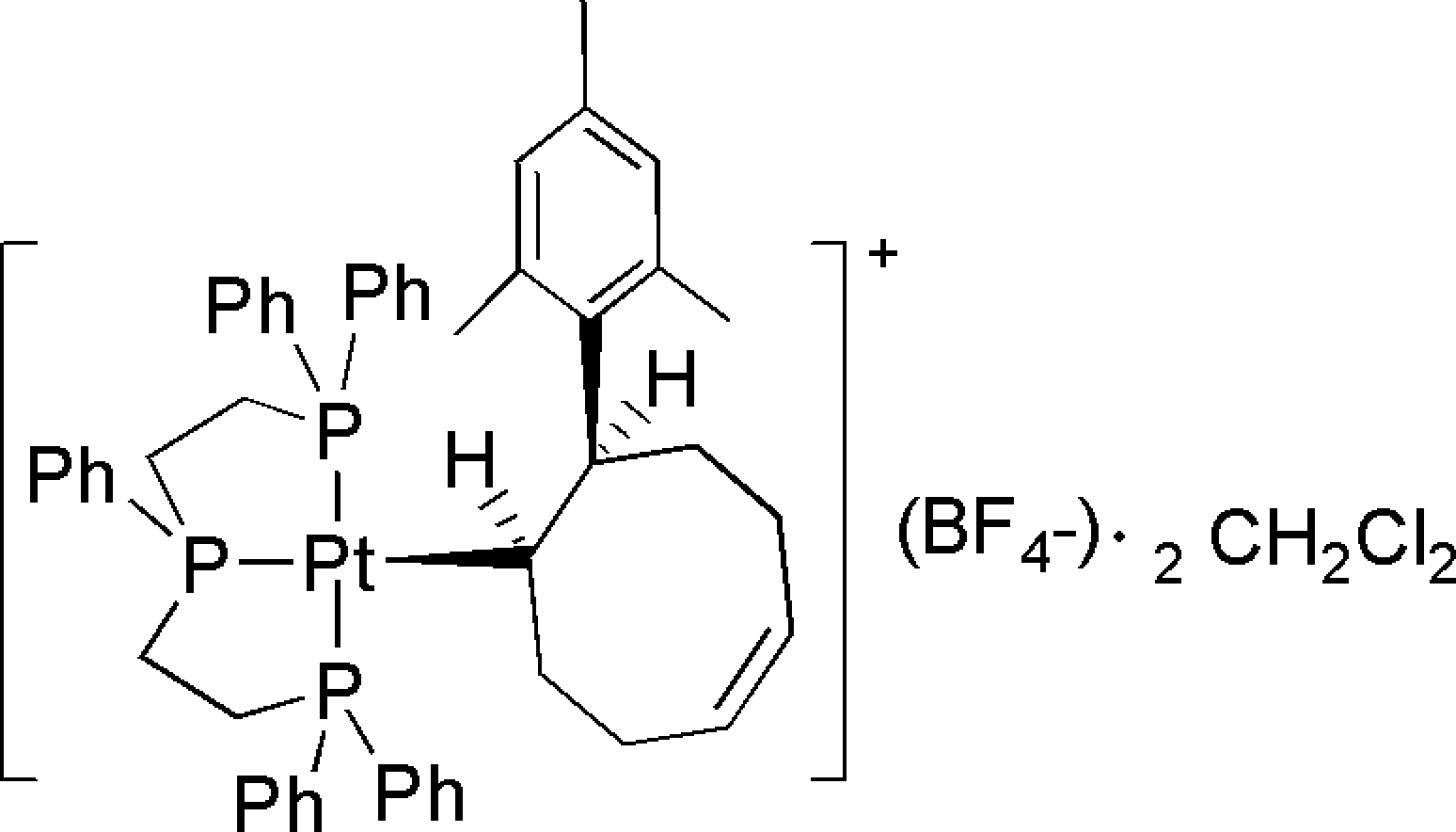


## Experimental

### 

#### Crystal data

[Pt(C_17_H_23_)(C_34_H_33_P_3_)](BF_4_)·2CH_2_Cl_2_
*M* *_r_* = 1213.62
Triclinic, 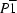

*a* = 10.1347 (2) Å
*b* = 14.0808 (3) Å
*c* = 19.8975 (4) Å
α = 69.485 (1)°
β = 77.798 (1)°
γ = 87.516 (1)°
*V* = 2597.84 (9) Å^3^
*Z* = 2
Mo *K*α radiation
μ = 3.05 mm^−1^
*T* = 180 K
0.25 × 0.15 × 0.10 mm

#### Data collection

Bruker APEXII CCD diffractometer
Absorption correction: multi-scan (*XSHELL*; Bruker, 1999) ▶ *T*_min_ = 0.516, *T*_max_ = 0.750
18596 measured reflections
10061 independent reflections
9410 reflections with *I* > 2σ(*I*)
*R*_int_ = 0.017

#### Refinement

*R*[*F* ^2^ > 2σ(*F* ^2^)] = 0.022
*wR*(*F* ^2^) = 0.053
*S* = 1.01
10061 reflections
655 parameters
H atoms treated by a mixture of independent and constrained refinement
Δρ_max_ = 0.85 e Å^−3^
Δρ_min_ = −0.69 e Å^−3^

### 

Data collection: *APEX2* (Bruker, 2005 ▶); cell refinement: *SAINT* (Bruker, 2005 ▶); data reduction: *SAINT*; program(s) used to solve structure: *XPREP* (Bruker, 2005 ▶) and *SHELXTL* (Sheldrick, 2008 ▶); program(s) used to refine structure: *SHELXTL*; molecular graphics: *SHELXTL*; software used to prepare material for publication: *SHELXTL*.

## Supplementary Material

Crystal structure: contains datablock(s) global, I. DOI: 10.1107/S1600536811023853/jh2297sup1.cif

Supplementary material file. DOI: 10.1107/S1600536811023853/jh2297Isup2.cdx

Structure factors: contains datablock(s) I. DOI: 10.1107/S1600536811023853/jh2297Isup3.hkl

Additional supplementary materials:  crystallographic information; 3D view; checkCIF report

## Figures and Tables

**Table:** 

Pt1—C1	2.166 (2)
Pt1—P3	2.2906 (7)
Pt1—P2	2.2995 (6)
Pt1—P1	2.3289 (6)

**Table:** 

C1—Pt1—P3	90.04 (7)
C1—Pt1—P2	174.01 (7)
P3—Pt1—P2	83.98 (2)
C1—Pt1—P1	102.75 (7)
P3—Pt1—P1	153.18 (2)
P2—Pt1—P1	82.86 (2)

**Fig. 1.**
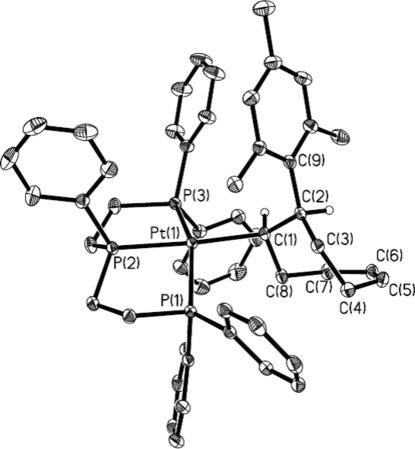
A drawing of the cationic moiety of the title compound (The solvent molecules, BF4- anion and H atoms except those on the C1 and C2 atoms are omitted for clarity; Displacement ellipsoids for non-H atoms are shown at the 30% probability level.)

**Fig. 2.**
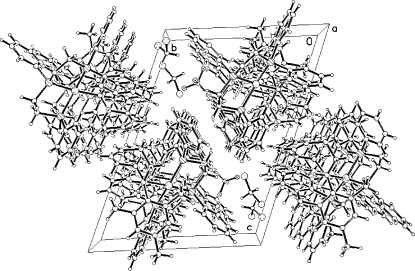
The packing of unit cell of the title compound, viewed down the a axis.

**Fig. 3.**
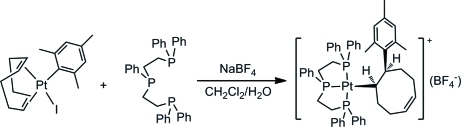
The formation of the title compound.

## supplementary crystallographic information

### Comment

Migratory insertion of metal-carbon (M—C) bond into alkenes is the cornerstone of many transition-metal catalyzed C—C bond forming processes such as Ziegler-Natta polymerization and the palladium catalyzed Heck type couplings. Mechanistically, a migratory insertion process involves incipient development of a bond between the metal and an alkene carbon *via* a planar four-center transition state, which qualitatively renders the *β*-carbon positively charged with the carbon bound to the metal being negatively charged, and subsequent carbon migration from the metal to the *β*-carbon to formally furnish both a new C—C and a new M—C bond. The strength of the M—C bond significantly affects the kinetics of the insertion process, with the reaction rate drastically decreasing with increasing M—C bond strength. M—C bonds for the third-row late transition metals especially Ir and Pt are reluctant towards migratory insertion reactions because of their high bond strength. In contrast to the ease of Ni—C and Pd—C in participating migratory insertions, to our knowledge, examples for their heavier congener Pt remain exceptionally rare, with the reaction generating the title compound herein representing a rather intriguing case of Pt—C migratory insertion reactions enabled by ligand coordination.

The structure of the cationic moiety of the title compound is shown in Fig. 1, with selected bond length and angles listed in Table 1. The Pt^II^ center is four-coordinate, with triphos acting as a tridentate ligand and the large 8-mesitylcyclooct-4*Z*-en-1-yl group occupying the 4t h coordination site of the Pt center. The Pt1—C1 bond is measured to be 2.166 (2) Å in length, similar to previously reported triphos-chelating Pt^II^-alkyl compounds [Koh *et al.* (2004); Feducia *et al.* (2008); Sokol *et al.* (2011)]. The three Pt—P bonds all show a bond length around 2.3 Å, with P2—Pt1—P1 and P3—Pt1—P2 bond angles being 82.86 (2)*^o^* and 83.98 (2)*^o^*, respectively. While the C1—C2 bond shows a length [1.553 (3) Å] common for a C—C single bond, the C5—C6 bond exhibits a length [1.328 (4) Å] most typical for a C=C double bond. It is also clear that the mesityl group and the Pt moiety are *cis*-to each other while both attaching to the cyclooct-4*Z*-ene motif. This configuration is in good agreement with the mechanistically predicted Pt-mesityl to COD migratory insertion product.

One of the unit cell packing diagrams for the title compound is shown in Fig. 2. The solvent molecules and the BF_4_^-^ anion reside in small cavities created by columns of the staked cationic Pt^II^ moiety.

### Experimental

The title compound was obtained unexpectedly while attempting to synthesize [(triphos)Pt-mesityl](BF_4_) *via* the ligand metathesis reaction between triphos and (COD)Pt(mesityl)(I): A mixture of triphos (0.15 g, 0.28 mmol) and (COD)Pt(mesityl)(I) (0.15 g, 0.27 mmol) in 5 ml dry CH_2_Cl_2_ was stirred under N_2_ for 2 h at room temperature. An aqueous solution of NaBF_4_ (0.25 g, 2.3 mmol, in 5 ml H_2_O) was added, the resulting mixture was stirred for 15 min. After separation of the organic layer, extraction with CH_2_Cl_2_ (5 ml *x* 2) and removal of the solvent, the residue was purified by flash chromatography on silica gel using CH_2_Cl_2_/MeNO_2_ (1: 1) as the eluent to afford the title compound as a white solid (40% yield). Colorless crystals were obtained by slow evaporation of a CH_2_Cl_2_/hexanes mixed solution.

### Refinement

All non-hydrogen atoms were refined anisotropically. The BF_4_^-^ anion was disordered, wherein the disordered fluoride atoms were refined in parts, each with their corresponding occupancy. The chloride atom of one methylene chloride molecule was disordered in two parts, each assigned 50% occupancy. The H atoms on C1 and C17 were located from difference Fourier maps and refined with H as riding atom (*U*_iso_ = 1.2(C)). All the other H atoms were placed in geometrically calculated positions, with C—H = 0.95 (aromatic), 0.99 (CH_2_), and 0.98 (CH_3_) Å, and refined as riding atoms, with *U*_iso_(H) = 1.5*U*_eq_C (CH_3_) or 1.2*U*_eq_C (other C), and the methyl groups were refined with AFIX 137, which allowed the rotation of the methyl groups whilst keeping the C—H distances and *X*—C—H angles fixed. The two hydrogen atoms of the disordered methylene chloride molecule were not added.

### Crystal data

**Table:**

[Pt(C_17_H_23_)(C_34_H_33_P_3_)](BF_4_)·2CH_2_Cl_2_	*Z* = 2
*M**_r_* = 1213.62	*F*(000) = 1220
Triclinic, *P*1	*D*_x_ = 1.551 Mg m^−^^3^
Hall symbol: -P 1	Mo *K*α radiation, λ = 0.71073 Å
*a* = 10.1347 (2) Å	Cell parameters from 9914 reflections
*b* = 14.0808 (3) Å	θ = 2.6–27.1°
*c* = 19.8975 (4) Å	µ = 3.05 mm^−^^1^
α = 69.485 (1)°	*T* = 180 K
β = 77.798 (1)°	Block, colourless
γ = 87.516 (1)°	0.25 × 0.15 × 0.10 mm
*V* = 2597.84 (9) Å^3^	

### Data collection

**Table:**

Bruker APEXII CCD diffractometer	10061 independent reflections
Radiation source: fine-focus sealed X-ray tube	9410 reflections with *I* > 2σ(*I*)
graphite	*R*_int_ = 0.017
φ and ω scans	θ_max_ = 26.0°, θ_min_ = 2.1°
Absorption correction: multi-scan (XSHELL; Bruker, 1999)	*h* = −12→12
*T*_min_ = 0.516, *T*_max_ = 0.750	*k* = −17→17
18596 measured reflections	*l* = −23→24

### Refinement

**Table:**

Refinement on *F*^2^	0 constraints
Least-squares matrix: full	H atoms treated by a mixture of independent and constrained refinement
*R*[*F*^2^ > 2σ(*F*^2^)] = 0.022	*w* = 1/[σ^2^(*F*_o_^2^) + (0.0239*P*)^2^ + 2.7018*P*] where *P* = (*F*_o_^2^ + 2*F*_c_^2^)/3
*wR*(*F*^2^) = 0.053	(Δ/σ)_max_ = 0.005
*S* = 1.01	Δρ_max_ = 0.85 e Å^−^^3^
10061 reflections	Δρ_min_ = −0.69 e Å^−^^3^
655 parameters	

### Special details

**Table:**

Geometry. All e.s.d.'s (except the e.s.d. in the dihedral angle between two l.s. planes) are estimated using the full covariance matrix. The cell e.s.d.'s are taken into account individually in the estimation of e.s.d.'s in distances, angles and torsion angles; correlations between e.s.d.'s in cell parameters are only used when they are defined by crystal symmetry. An approximate (isotropic) treatment of cell e.s.d.'s is used for estimating e.s.d.'s involving l.s. planes.
Refinement. Refinement of *F*^2^ against ALL reflections. The weighted *R*-factor *wR* and goodness of fit *S* are based on *F*^2^, conventional *R*-factors *R* are based on *F*, with *F* set to zero for negative *F*^2^. The threshold expression of *F*^2^ > 2*σ*(*F*^2^) is used only for calculating *R*-factors(gt) *etc*. and is not relevant to the choice of reflections for refinement. *R*-factors based on *F*^2^ are statistically about twice as large as those based on *F*, and *R*-factors based on ALL data will be even larger.

### Fractional atomic coordinates and isotropic or equivalent isotropic displacement parameters (Å^2^)

**Table:**

	*x*	*y*	*z*	*U*_iso_*/*U*_eq_	Occ. (<1)
Pt1	0.364017 (9)	0.651864 (7)	0.711204 (5)	0.01904 (4)	
P1	0.42946 (6)	0.61844 (5)	0.60245 (4)	0.02073 (13)	
P2	0.22872 (7)	0.50870 (5)	0.74564 (4)	0.02289 (13)	
P3	0.20832 (6)	0.68965 (5)	0.79913 (4)	0.02262 (13)	
C1	0.4774 (3)	0.79079 (19)	0.68775 (14)	0.0221 (5)	
H1	0.441 (3)	0.813 (2)	0.7254 (17)	0.026*	
C2	0.6308 (2)	0.79102 (19)	0.68731 (14)	0.0224 (5)	
H2A	0.6514	0.8629	0.6809	0.027*	
C3	0.7286 (3)	0.7752 (2)	0.62203 (14)	0.0259 (5)	
H3A	0.7007	0.7118	0.6171	0.031*	
H3B	0.8200	0.7656	0.6332	0.031*	
C4	0.7375 (3)	0.8624 (2)	0.54734 (15)	0.0302 (6)	
H4A	0.8211	0.8553	0.5135	0.036*	
H4B	0.6603	0.8541	0.5264	0.036*	
C5	0.7376 (3)	0.9684 (2)	0.54929 (16)	0.0339 (6)	
H5A	0.8217	1.0051	0.5307	0.041*	
C6	0.6334 (3)	1.0164 (2)	0.57396 (16)	0.0335 (6)	
H6A	0.6545	1.0831	0.5707	0.040*	
C7	0.4881 (3)	0.9829 (2)	0.60610 (17)	0.0335 (6)	
H7A	0.4340	1.0293	0.5730	0.040*	
H7B	0.4638	0.9948	0.6533	0.040*	
C8	0.4391 (3)	0.87384 (19)	0.62101 (16)	0.0271 (6)	
H8A	0.3395	0.8728	0.6279	0.033*	
H8B	0.4760	0.8562	0.5768	0.033*	
C9	0.6651 (2)	0.7307 (2)	0.76218 (14)	0.0242 (5)	
C10	0.6741 (3)	0.7831 (2)	0.80972 (15)	0.0297 (6)	
C11	0.7080 (3)	0.7320 (2)	0.87765 (16)	0.0349 (7)	
H11A	0.7169	0.7693	0.9081	0.042*	
C12	0.7289 (3)	0.6290 (2)	0.90191 (15)	0.0342 (7)	
C13	0.7188 (3)	0.5779 (2)	0.85512 (15)	0.0312 (6)	
H13A	0.7319	0.5069	0.8709	0.037*	
C14	0.6903 (3)	0.6261 (2)	0.78569 (15)	0.0257 (5)	
C15	0.6463 (4)	0.8949 (2)	0.79106 (19)	0.0422 (8)	
H15A	0.6563	0.9160	0.8318	0.063*	
H15B	0.5541	0.9068	0.7829	0.063*	
H15C	0.7106	0.9341	0.7465	0.063*	
C16	0.7627 (4)	0.5752 (3)	0.97645 (17)	0.0485 (8)	
H16A	0.7730	0.5028	0.9843	0.073*	
H16B	0.6897	0.5837	1.0147	0.073*	
H16C	0.8473	0.6042	0.9785	0.073*	
C17	0.6909 (3)	0.5620 (2)	0.73902 (17)	0.0293 (6)	
H17A	0.618 (3)	0.578 (2)	0.7116 (16)	0.030 (8)*	
H17B	0.771 (3)	0.576 (2)	0.7019 (18)	0.037 (8)*	
H17C	0.688 (3)	0.489 (3)	0.7679 (19)	0.044 (9)*	
C18	0.3175 (3)	0.68603 (19)	0.54202 (15)	0.0252 (5)	
C19	0.2125 (3)	0.7407 (2)	0.56691 (16)	0.0289 (6)	
H19A	0.2067	0.7499	0.6125	0.035*	
C20	0.1164 (3)	0.7816 (2)	0.52517 (18)	0.0365 (7)	
H20A	0.0444	0.8182	0.5425	0.044*	
C21	0.1251 (3)	0.7695 (2)	0.45872 (18)	0.0407 (8)	
H21A	0.0587	0.7973	0.4306	0.049*	
C22	0.2300 (3)	0.7168 (2)	0.43277 (17)	0.0400 (7)	
H22A	0.2369	0.7100	0.3863	0.048*	
C23	0.3246 (3)	0.6742 (2)	0.47465 (16)	0.0317 (6)	
H23A	0.3952	0.6365	0.4574	0.038*	
C24	0.5955 (3)	0.6261 (2)	0.54362 (14)	0.0239 (5)	
C25	0.6344 (3)	0.7048 (2)	0.47624 (15)	0.0279 (6)	
H25A	0.5732	0.7566	0.4599	0.034*	
C26	0.7627 (3)	0.7073 (2)	0.43301 (16)	0.0345 (7)	
H26A	0.7886	0.7608	0.3872	0.041*	
C27	0.8521 (3)	0.6326 (2)	0.45636 (17)	0.0364 (7)	
H27A	0.9389	0.6342	0.4263	0.044*	
C28	0.8159 (3)	0.5556 (2)	0.52315 (18)	0.0350 (7)	
H28A	0.8785	0.5050	0.5395	0.042*	
C29	0.6880 (3)	0.5517 (2)	0.56672 (15)	0.0277 (6)	
H29A	0.6634	0.4980	0.6125	0.033*	
C30	0.3756 (3)	0.4858 (2)	0.62265 (15)	0.0266 (6)	
H30A	0.3790	0.4736	0.5762	0.032*	
H30B	0.4374	0.4387	0.6499	0.032*	
C31	0.2312 (3)	0.4662 (2)	0.66878 (15)	0.0278 (6)	
H31A	0.2060	0.3931	0.6868	0.033*	
H31B	0.1665	0.5045	0.6391	0.033*	
C32	0.2708 (3)	0.3995 (2)	0.81810 (15)	0.0270 (6)	
C33	0.3937 (3)	0.3991 (3)	0.83828 (18)	0.0408 (7)	
H33A	0.4525	0.4575	0.8161	0.049*	
C34	0.4312 (4)	0.3140 (3)	0.8906 (2)	0.0556 (10)	
H34A	0.5169	0.3132	0.9030	0.067*	
C35	0.3443 (4)	0.2307 (3)	0.92476 (19)	0.0538 (10)	
H35A	0.3689	0.1729	0.9616	0.065*	
C36	0.2227 (4)	0.2311 (3)	0.9056 (2)	0.0518 (9)	
H36A	0.1629	0.1734	0.9296	0.062*	
C37	0.1850 (3)	0.3137 (2)	0.85200 (19)	0.0419 (7)	
H37A	0.1011	0.3122	0.8383	0.050*	
C38	0.0578 (3)	0.5398 (2)	0.78013 (17)	0.0305 (6)	
H38A	0.0182	0.5847	0.7392	0.037*	
H38B	0.0001	0.4773	0.8055	0.037*	
C39	0.0671 (3)	0.5937 (2)	0.83370 (16)	0.0306 (6)	
H39A	0.0790	0.5426	0.8808	0.037*	
H39B	−0.0189	0.6275	0.8433	0.037*	
C40	0.1355 (3)	0.8110 (2)	0.75747 (15)	0.0269 (6)	
C41	0.0349 (3)	0.8185 (2)	0.71767 (17)	0.0360 (7)	
H41A	0.0000	0.7591	0.7143	0.043*	
C42	−0.0139 (4)	0.9122 (3)	0.6832 (2)	0.0483 (8)	
H42A	−0.0815	0.9173	0.6557	0.058*	
C43	0.0352 (4)	0.9987 (3)	0.6887 (2)	0.0534 (9)	
H43A	0.0009	1.0629	0.6651	0.064*	
C44	0.1336 (3)	0.9921 (2)	0.7284 (2)	0.0461 (8)	
H44A	0.1667	1.0516	0.7324	0.055*	
C45	0.1838 (3)	0.8990 (2)	0.76225 (17)	0.0341 (6)	
H45A	0.2522	0.8948	0.7892	0.041*	
C46	0.2372 (3)	0.6987 (2)	0.88357 (15)	0.0271 (6)	
C47	0.3603 (3)	0.6794 (3)	0.90346 (16)	0.0360 (7)	
H47A	0.4345	0.6626	0.8721	0.043*	
C48	0.3752 (3)	0.6848 (3)	0.96998 (19)	0.0516 (9)	
H48A	0.4602	0.6717	0.9838	0.062*	
C49	0.2688 (4)	0.7088 (3)	1.01592 (19)	0.0529 (9)	
H49A	0.2802	0.7123	1.0612	0.064*	
C50	0.1450 (3)	0.7277 (3)	0.99613 (18)	0.0478 (9)	
H50A	0.0708	0.7436	1.0281	0.057*	
C51	0.1286 (3)	0.7238 (3)	0.93033 (17)	0.0406 (7)	
H51A	0.0437	0.7380	0.9165	0.049*	
C52	0.4898 (4)	0.1369 (3)	0.7821 (2)	0.0539 (9)	
H52A	0.5104	0.1828	0.8068	0.065*	
H52B	0.5751	0.1061	0.7664	0.065*	
C53	0.8544 (7)	0.0658 (5)	0.8821 (4)	0.133 (3)	
Cl1	0.37441 (11)	0.04042 (7)	0.84446 (6)	0.0616 (2)	
Cl2	0.42619 (14)	0.20709 (9)	0.70494 (6)	0.0783 (3)	
Cl4	0.68482 (18)	0.04661 (11)	0.93024 (9)	0.1038 (5)	
Cl3A	0.9758 (7)	−0.0097 (5)	0.9226 (4)	0.136 (2)	0.50
Cl3B	0.9361 (5)	−0.0391 (4)	0.9105 (3)	0.1094 (18)	0.50
B1	0.8670 (5)	0.3112 (3)	0.7387 (2)	0.0478 (10)	
F1	0.9055 (3)	0.40045 (19)	0.68343 (13)	0.0780 (8)	
F2	0.8778 (3)	0.3169 (2)	0.80450 (13)	0.0756 (7)	
F3A	0.9454 (17)	0.2390 (16)	0.7191 (13)	0.073 (7)	0.50
F4A	0.736 (2)	0.2949 (17)	0.7474 (12)	0.116 (9)	0.50
F4B	0.773 (2)	0.2428 (19)	0.7456 (9)	0.085 (9)	0.25
F3B	1.001 (2)	0.2496 (18)	0.7311 (11)	0.059 (4)	0.25
F4C	0.718 (3)	0.321 (2)	0.7325 (14)	0.053 (4)	0.25
F3C	0.892 (3)	0.229 (3)	0.724 (2)	0.063 (10)	0.25

### Atomic displacement parameters (Å^2^)

**Table:**

	*U*^11^	*U*^22^	*U*^33^	*U*^12^	*U*^13^	*U*^23^
Pt1	0.01793 (5)	0.01961 (5)	0.02047 (5)	−0.00080 (3)	−0.00418 (4)	−0.00782 (4)
P1	0.0213 (3)	0.0208 (3)	0.0212 (3)	−0.0009 (2)	−0.0047 (3)	−0.0083 (3)
P2	0.0208 (3)	0.0230 (3)	0.0253 (3)	−0.0031 (2)	−0.0028 (3)	−0.0097 (3)
P3	0.0196 (3)	0.0257 (3)	0.0247 (3)	0.0008 (3)	−0.0041 (3)	−0.0117 (3)
C1	0.0222 (13)	0.0201 (12)	0.0254 (13)	−0.0010 (10)	−0.0051 (10)	−0.0096 (10)
C2	0.0209 (12)	0.0213 (12)	0.0273 (13)	−0.0019 (10)	−0.0075 (10)	−0.0096 (10)
C3	0.0226 (13)	0.0273 (13)	0.0285 (14)	−0.0016 (10)	−0.0059 (11)	−0.0102 (11)
C4	0.0284 (14)	0.0328 (15)	0.0290 (14)	−0.0009 (11)	−0.0059 (11)	−0.0100 (12)
C5	0.0337 (15)	0.0294 (15)	0.0323 (15)	−0.0088 (12)	−0.0079 (12)	−0.0013 (12)
C6	0.0378 (16)	0.0211 (13)	0.0392 (16)	−0.0064 (12)	−0.0131 (13)	−0.0035 (12)
C7	0.0341 (15)	0.0214 (13)	0.0426 (17)	0.0012 (11)	−0.0113 (13)	−0.0064 (12)
C8	0.0224 (13)	0.0232 (13)	0.0357 (15)	−0.0009 (10)	−0.0099 (11)	−0.0078 (12)
C9	0.0173 (12)	0.0292 (14)	0.0267 (13)	−0.0004 (10)	−0.0049 (10)	−0.0103 (11)
C10	0.0261 (14)	0.0361 (15)	0.0302 (14)	0.0023 (11)	−0.0071 (11)	−0.0153 (12)
C11	0.0277 (14)	0.0538 (19)	0.0306 (15)	0.0048 (13)	−0.0087 (12)	−0.0226 (14)
C12	0.0210 (13)	0.0525 (19)	0.0258 (14)	0.0046 (12)	−0.0046 (11)	−0.0104 (13)
C13	0.0227 (13)	0.0359 (15)	0.0306 (15)	0.0059 (11)	−0.0063 (11)	−0.0063 (12)
C14	0.0178 (12)	0.0288 (14)	0.0295 (14)	0.0010 (10)	−0.0037 (10)	−0.0097 (11)
C15	0.054 (2)	0.0409 (18)	0.0455 (18)	0.0078 (15)	−0.0188 (16)	−0.0277 (15)
C16	0.0440 (19)	0.067 (2)	0.0310 (17)	0.0090 (17)	−0.0102 (14)	−0.0120 (16)
C17	0.0277 (15)	0.0259 (14)	0.0361 (16)	0.0049 (11)	−0.0101 (13)	−0.0117 (12)
C18	0.0245 (13)	0.0233 (13)	0.0279 (13)	−0.0049 (10)	−0.0093 (11)	−0.0062 (11)
C19	0.0249 (13)	0.0285 (14)	0.0301 (14)	−0.0042 (11)	−0.0069 (11)	−0.0048 (11)
C20	0.0260 (14)	0.0308 (15)	0.0473 (18)	−0.0015 (12)	−0.0140 (13)	−0.0030 (13)
C21	0.0384 (17)	0.0337 (16)	0.0470 (19)	−0.0077 (13)	−0.0265 (15)	0.0006 (14)
C22	0.0501 (19)	0.0383 (17)	0.0349 (16)	−0.0088 (14)	−0.0200 (15)	−0.0094 (14)
C23	0.0370 (16)	0.0305 (15)	0.0303 (15)	−0.0040 (12)	−0.0115 (12)	−0.0107 (12)
C24	0.0235 (13)	0.0265 (13)	0.0252 (13)	−0.0030 (10)	−0.0046 (10)	−0.0132 (11)
C25	0.0303 (14)	0.0284 (14)	0.0278 (14)	−0.0034 (11)	−0.0072 (11)	−0.0118 (11)
C26	0.0343 (16)	0.0421 (17)	0.0273 (14)	−0.0148 (13)	0.0014 (12)	−0.0150 (13)
C27	0.0260 (14)	0.0503 (19)	0.0406 (17)	−0.0085 (13)	0.0012 (13)	−0.0287 (15)
C28	0.0256 (14)	0.0413 (17)	0.0468 (18)	0.0024 (12)	−0.0072 (13)	−0.0263 (15)
C29	0.0285 (14)	0.0293 (14)	0.0290 (14)	−0.0005 (11)	−0.0051 (11)	−0.0151 (12)
C30	0.0301 (14)	0.0242 (13)	0.0272 (14)	−0.0020 (11)	−0.0037 (11)	−0.0120 (11)
C31	0.0304 (14)	0.0261 (14)	0.0289 (14)	−0.0057 (11)	−0.0052 (11)	−0.0118 (11)
C32	0.0275 (14)	0.0276 (14)	0.0246 (13)	0.0001 (11)	−0.0006 (11)	−0.0102 (11)
C33	0.0376 (17)	0.0434 (18)	0.0396 (17)	−0.0019 (14)	−0.0121 (14)	−0.0095 (14)
C34	0.057 (2)	0.065 (2)	0.047 (2)	0.0164 (19)	−0.0282 (18)	−0.0140 (19)
C35	0.076 (3)	0.045 (2)	0.0346 (18)	0.0196 (19)	−0.0116 (18)	−0.0080 (15)
C36	0.060 (2)	0.0298 (17)	0.047 (2)	0.0019 (15)	0.0071 (17)	−0.0016 (15)
C37	0.0360 (17)	0.0326 (16)	0.0477 (19)	−0.0037 (13)	0.0001 (14)	−0.0071 (14)
C38	0.0209 (13)	0.0307 (14)	0.0414 (16)	−0.0024 (11)	−0.0040 (12)	−0.0155 (13)
C39	0.0222 (13)	0.0315 (15)	0.0361 (15)	−0.0027 (11)	0.0015 (11)	−0.0131 (12)
C40	0.0241 (13)	0.0302 (14)	0.0273 (14)	0.0046 (11)	−0.0058 (11)	−0.0113 (11)
C41	0.0313 (15)	0.0393 (17)	0.0430 (17)	0.0066 (13)	−0.0147 (13)	−0.0180 (14)
C42	0.0439 (19)	0.053 (2)	0.054 (2)	0.0157 (16)	−0.0250 (17)	−0.0181 (17)
C43	0.060 (2)	0.0373 (18)	0.058 (2)	0.0160 (16)	−0.0229 (19)	−0.0054 (16)
C44	0.0474 (19)	0.0296 (16)	0.060 (2)	0.0017 (14)	−0.0139 (17)	−0.0121 (15)
C45	0.0308 (15)	0.0337 (15)	0.0403 (17)	0.0033 (12)	−0.0097 (13)	−0.0147 (13)
C46	0.0262 (13)	0.0321 (14)	0.0251 (13)	0.0025 (11)	−0.0056 (11)	−0.0126 (11)
C47	0.0254 (14)	0.057 (2)	0.0309 (15)	0.0034 (13)	−0.0047 (12)	−0.0226 (14)
C48	0.0297 (16)	0.097 (3)	0.0416 (19)	0.0082 (17)	−0.0117 (14)	−0.038 (2)
C49	0.048 (2)	0.089 (3)	0.0355 (18)	0.0103 (19)	−0.0132 (16)	−0.0370 (19)
C50	0.0402 (18)	0.075 (2)	0.0351 (17)	0.0151 (17)	−0.0045 (14)	−0.0312 (17)
C51	0.0294 (15)	0.061 (2)	0.0366 (17)	0.0130 (14)	−0.0082 (13)	−0.0247 (16)
C52	0.057 (2)	0.050 (2)	0.054 (2)	0.0027 (17)	−0.0147 (18)	−0.0151 (18)
C53	0.116 (5)	0.085 (4)	0.142 (6)	−0.015 (4)	−0.023 (5)	0.026 (4)
Cl1	0.0693 (6)	0.0494 (5)	0.0631 (6)	−0.0019 (4)	0.0001 (5)	−0.0236 (5)
Cl2	0.0985 (9)	0.0721 (7)	0.0616 (6)	−0.0003 (6)	−0.0329 (6)	−0.0105 (5)
Cl4	0.1268 (13)	0.0818 (9)	0.0925 (10)	−0.0043 (8)	−0.0232 (9)	−0.0167 (8)
Cl3A	0.125 (3)	0.149 (6)	0.100 (3)	−0.026 (3)	−0.029 (3)	0.005 (3)
Cl3B	0.137 (4)	0.077 (2)	0.079 (3)	0.017 (3)	0.027 (3)	−0.0152 (17)
B1	0.059 (3)	0.050 (2)	0.035 (2)	−0.010 (2)	−0.0051 (18)	−0.0167 (18)
F1	0.0911 (19)	0.0644 (15)	0.0529 (14)	0.0143 (13)	0.0148 (13)	−0.0077 (12)
F2	0.107 (2)	0.0828 (17)	0.0497 (13)	−0.0007 (15)	−0.0303 (13)	−0.0300 (12)
F3A	0.086 (19)	0.058 (5)	0.075 (6)	0.021 (12)	−0.018 (11)	−0.024 (4)
F4A	0.057 (8)	0.17 (2)	0.131 (14)	−0.045 (11)	−0.005 (8)	−0.071 (13)
F4B	0.089 (19)	0.121 (18)	0.042 (7)	−0.062 (15)	−0.020 (9)	−0.014 (9)
F3B	0.050 (11)	0.062 (8)	0.059 (9)	0.012 (7)	−0.007 (7)	−0.017 (6)
F4C	0.050 (9)	0.073 (9)	0.058 (7)	−0.003 (6)	−0.023 (6)	−0.043 (7)
F3C	0.08 (2)	0.058 (14)	0.073 (16)	0.041 (17)	−0.040 (18)	−0.044 (14)

### Geometric parameters (Å, °)

**Table:**

Pt1—C1	2.166 (2)	C25—C26	1.393 (4)
Pt1—P3	2.2906 (7)	C25—H25A	0.9500
Pt1—P2	2.2995 (6)	C26—C27	1.377 (4)
Pt1—P1	2.3289 (6)	C26—H26A	0.9500
P1—C24	1.819 (3)	C27—C28	1.376 (4)
P1—C18	1.822 (3)	C27—H27A	0.9500
P1—C30	1.849 (3)	C28—C29	1.390 (4)
P2—C32	1.810 (3)	C28—H28A	0.9500
P2—C38	1.818 (3)	C29—H29A	0.9500
P2—C31	1.822 (3)	C30—C31	1.533 (4)
P3—C46	1.813 (3)	C30—H30A	0.9900
P3—C40	1.819 (3)	C30—H30B	0.9900
P3—C39	1.856 (3)	C31—H31A	0.9900
C1—C8	1.540 (4)	C31—H31B	0.9900
C1—C2	1.553 (3)	C32—C33	1.387 (4)
C1—H1	0.91 (3)	C32—C37	1.393 (4)
C2—C3	1.536 (4)	C33—C34	1.385 (5)
C2—C9	1.539 (4)	C33—H33A	0.9500
C2—H2A	1.0000	C34—C35	1.376 (6)
C3—C4	1.549 (4)	C34—H34A	0.9500
C3—H3A	0.9900	C35—C36	1.364 (6)
C3—H3B	0.9900	C35—H35A	0.9500
C4—C5	1.506 (4)	C36—C37	1.379 (5)
C4—H4A	0.9900	C36—H36A	0.9500
C4—H4B	0.9900	C37—H37A	0.9500
C5—C6	1.328 (4)	C38—C39	1.529 (4)
C5—H5A	0.9500	C38—H38A	0.9900
C6—C7	1.501 (4)	C38—H38B	0.9900
C6—H6A	0.9500	C39—H39A	0.9900
C7—C8	1.542 (4)	C39—H39B	0.9900
C7—H7A	0.9900	C40—C45	1.392 (4)
C7—H7B	0.9900	C40—C41	1.397 (4)
C8—H8A	0.9900	C41—C42	1.382 (4)
C8—H8B	0.9900	C41—H41A	0.9500
C9—C10	1.407 (4)	C42—C43	1.382 (5)
C9—C14	1.410 (4)	C42—H42A	0.9500
C10—C11	1.399 (4)	C43—C44	1.378 (5)
C10—C15	1.513 (4)	C43—H43A	0.9500
C11—C12	1.381 (4)	C44—C45	1.379 (4)
C11—H11A	0.9500	C44—H44A	0.9500
C12—C13	1.382 (4)	C45—H45A	0.9500
C12—C16	1.515 (4)	C46—C47	1.375 (4)
C13—C14	1.395 (4)	C46—C51	1.401 (4)
C13—H13A	0.9500	C47—C48	1.391 (4)
C14—C17	1.504 (4)	C47—H47A	0.9500
C15—H15A	0.9800	C48—C49	1.371 (5)
C15—H15B	0.9800	C48—H48A	0.9500
C15—H15C	0.9800	C49—C50	1.381 (5)
C16—H16A	0.9800	C49—H49A	0.9500
C16—H16B	0.9800	C50—C51	1.374 (4)
C16—H16C	0.9800	C50—H50A	0.9500
C17—H17A	0.98 (3)	C51—H51A	0.9500
C17—H17B	0.95 (3)	C52—Cl2	1.746 (4)
C17—H17C	0.99 (3)	C52—Cl1	1.760 (4)
C18—C19	1.393 (4)	C52—H52A	0.9900
C18—C23	1.394 (4)	C52—H52B	0.9900
C19—C20	1.389 (4)	C53—Cl3B	1.641 (9)
C19—H19A	0.9500	C53—Cl3A	1.731 (10)
C20—C21	1.376 (5)	C53—Cl4	1.763 (7)
C20—H20A	0.9500	B1—F3C	1.29 (4)
C21—C22	1.384 (5)	B1—F4A	1.32 (2)
C21—H21A	0.9500	B1—F4B	1.34 (2)
C22—C23	1.383 (4)	B1—F1	1.354 (5)
C22—H22A	0.9500	B1—F2	1.368 (4)
C23—H23A	0.9500	B1—F3A	1.376 (19)
C24—C29	1.396 (4)	B1—F4C	1.53 (3)
C24—C25	1.397 (4)	B1—F3B	1.59 (2)
			
C1—Pt1—P3	90.04 (7)	C25—C24—P1	122.2 (2)
C1—Pt1—P2	174.01 (7)	C26—C25—C24	120.1 (3)
P3—Pt1—P2	83.98 (2)	C26—C25—H25A	119.9
C1—Pt1—P1	102.75 (7)	C24—C25—H25A	119.9
P3—Pt1—P1	153.18 (2)	C27—C26—C25	120.3 (3)
P2—Pt1—P1	82.86 (2)	C27—C26—H26A	119.8
C24—P1—C18	106.08 (12)	C25—C26—H26A	119.8
C24—P1—C30	101.49 (12)	C28—C27—C26	120.1 (3)
C18—P1—C30	100.41 (12)	C28—C27—H27A	119.9
C24—P1—Pt1	130.38 (8)	C26—C27—H27A	119.9
C18—P1—Pt1	107.54 (9)	C27—C28—C29	120.2 (3)
C30—P1—Pt1	107.03 (9)	C27—C28—H28A	119.9
C32—P2—C38	106.12 (13)	C29—C28—H28A	119.9
C32—P2—C31	105.75 (13)	C28—C29—C24	120.5 (3)
C38—P2—C31	109.85 (13)	C28—C29—H29A	119.8
C32—P2—Pt1	115.85 (9)	C24—C29—H29A	119.8
C38—P2—Pt1	107.51 (9)	C31—C30—P1	109.29 (18)
C31—P2—Pt1	111.56 (9)	C31—C30—H30A	109.8
C46—P3—C40	102.55 (12)	P1—C30—H30A	109.8
C46—P3—C39	101.85 (13)	C31—C30—H30B	109.8
C40—P3—C39	106.10 (13)	P1—C30—H30B	109.8
C46—P3—Pt1	126.74 (9)	H30A—C30—H30B	108.3
C40—P3—Pt1	109.43 (9)	C30—C31—P2	106.77 (18)
C39—P3—Pt1	108.43 (9)	C30—C31—H31A	110.4
C8—C1—C2	113.4 (2)	P2—C31—H31A	110.4
C8—C1—Pt1	109.26 (16)	C30—C31—H31B	110.4
C2—C1—Pt1	121.68 (17)	P2—C31—H31B	110.4
C8—C1—H1	102.5 (18)	H31A—C31—H31B	108.6
C2—C1—H1	102.5 (18)	C33—C32—C37	119.1 (3)
Pt1—C1—H1	105.0 (18)	C33—C32—P2	119.4 (2)
C3—C2—C9	113.8 (2)	C37—C32—P2	121.5 (2)
C3—C2—C1	117.6 (2)	C34—C33—C32	120.3 (3)
C9—C2—C1	113.7 (2)	C34—C33—H33A	119.9
C3—C2—H2A	103.0	C32—C33—H33A	119.9
C9—C2—H2A	103.0	C35—C34—C33	120.0 (3)
C1—C2—H2A	103.0	C35—C34—H34A	120.0
C2—C3—C4	115.4 (2)	C33—C34—H34A	120.0
C2—C3—H3A	108.4	C36—C35—C34	119.8 (3)
C4—C3—H3A	108.4	C36—C35—H35A	120.1
C2—C3—H3B	108.4	C34—C35—H35A	120.1
C4—C3—H3B	108.4	C35—C36—C37	121.1 (3)
H3A—C3—H3B	107.5	C35—C36—H36A	119.4
C5—C4—C3	116.0 (2)	C37—C36—H36A	119.4
C5—C4—H4A	108.3	C36—C37—C32	119.6 (3)
C3—C4—H4A	108.3	C36—C37—H37A	120.2
C5—C4—H4B	108.3	C32—C37—H37A	120.2
C3—C4—H4B	108.3	C39—C38—P2	107.08 (18)
H4A—C4—H4B	107.4	C39—C38—H38A	110.3
C6—C5—C4	127.5 (3)	P2—C38—H38A	110.3
C6—C5—H5A	116.3	C39—C38—H38B	110.3
C4—C5—H5A	116.3	P2—C38—H38B	110.3
C5—C6—C7	130.8 (3)	H38A—C38—H38B	108.6
C5—C6—H6A	114.6	C38—C39—P3	113.01 (19)
C7—C6—H6A	114.6	C38—C39—H39A	109.0
C6—C7—C8	121.3 (2)	P3—C39—H39A	109.0
C6—C7—H7A	107.0	C38—C39—H39B	109.0
C8—C7—H7A	107.0	P3—C39—H39B	109.0
C6—C7—H7B	107.0	H39A—C39—H39B	107.8
C8—C7—H7B	107.0	C45—C40—C41	118.9 (3)
H7A—C7—H7B	106.8	C45—C40—P3	120.1 (2)
C1—C8—C7	116.1 (2)	C41—C40—P3	121.0 (2)
C1—C8—H8A	108.3	C42—C41—C40	119.9 (3)
C7—C8—H8A	108.3	C42—C41—H41A	120.0
C1—C8—H8B	108.3	C40—C41—H41A	120.0
C7—C8—H8B	108.3	C41—C42—C43	120.3 (3)
H8A—C8—H8B	107.4	C41—C42—H42A	119.8
C10—C9—C14	117.8 (2)	C43—C42—H42A	119.8
C10—C9—C2	118.2 (2)	C44—C43—C42	120.2 (3)
C14—C9—C2	123.9 (2)	C44—C43—H43A	119.9
C11—C10—C9	120.3 (3)	C42—C43—H43A	119.9
C11—C10—C15	117.2 (3)	C43—C44—C45	119.8 (3)
C9—C10—C15	122.5 (3)	C43—C44—H44A	120.1
C12—C11—C10	122.2 (3)	C45—C44—H44A	120.1
C12—C11—H11A	118.9	C44—C45—C40	120.8 (3)
C10—C11—H11A	118.9	C44—C45—H45A	119.6
C11—C12—C13	117.1 (3)	C40—C45—H45A	119.6
C11—C12—C16	121.0 (3)	C47—C46—C51	119.7 (3)
C13—C12—C16	121.8 (3)	C47—C46—P3	122.1 (2)
C12—C13—C14	122.9 (3)	C51—C46—P3	118.2 (2)
C12—C13—H13A	118.5	C46—C47—C48	119.4 (3)
C14—C13—H13A	118.5	C46—C47—H47A	120.3
C13—C14—C9	119.6 (3)	C48—C47—H47A	120.3
C13—C14—C17	117.0 (2)	C49—C48—C47	120.9 (3)
C9—C14—C17	123.4 (2)	C49—C48—H48A	119.5
C10—C15—H15A	109.5	C47—C48—H48A	119.5
C10—C15—H15B	109.5	C48—C49—C50	119.7 (3)
H15A—C15—H15B	109.5	C48—C49—H49A	120.1
C10—C15—H15C	109.5	C50—C49—H49A	120.1
H15A—C15—H15C	109.5	C51—C50—C49	120.2 (3)
H15B—C15—H15C	109.5	C51—C50—H50A	119.9
C12—C16—H16A	109.5	C49—C50—H50A	119.9
C12—C16—H16B	109.5	C50—C51—C46	120.0 (3)
H16A—C16—H16B	109.5	C50—C51—H51A	120.0
C12—C16—H16C	109.5	C46—C51—H51A	120.0
H16A—C16—H16C	109.5	Cl2—C52—Cl1	111.7 (2)
H16B—C16—H16C	109.5	Cl2—C52—H52A	109.3
C14—C17—H17A	112.7 (17)	Cl1—C52—H52A	109.3
C14—C17—H17B	110.3 (19)	Cl2—C52—H52B	109.3
H17A—C17—H17B	104 (3)	Cl1—C52—H52B	109.3
C14—C17—H17C	112 (2)	H52A—C52—H52B	107.9
H17A—C17—H17C	110 (3)	Cl3B—C53—Cl4	109.6 (4)
H17B—C17—H17C	107 (3)	Cl3A—C53—Cl4	118.8 (4)
C19—C18—C23	118.9 (3)	F3C—B1—F1	117 (2)
C19—C18—P1	119.6 (2)	F4A—B1—F1	109.1 (10)
C23—C18—P1	121.0 (2)	F4B—B1—F1	129.0 (9)
C20—C19—C18	120.1 (3)	F3C—B1—F2	122 (2)
C20—C19—H19A	120.0	F4A—B1—F2	103.0 (9)
C18—C19—H19A	120.0	F4B—B1—F2	112.8 (8)
C21—C20—C19	120.2 (3)	F1—B1—F2	110.9 (3)
C21—C20—H20A	119.9	F4A—B1—F3A	113.7 (10)
C19—C20—H20A	119.9	F1—B1—F3A	105.9 (10)
C20—C21—C22	120.3 (3)	F2—B1—F3A	114.3 (10)
C20—C21—H21A	119.9	F3C—B1—F4C	98.9 (18)
C22—C21—H21A	119.9	F1—B1—F4C	93.6 (11)
C23—C22—C21	119.8 (3)	F2—B1—F4C	109.0 (9)
C23—C22—H22A	120.1	F3A—B1—F4C	121.0 (11)
C21—C22—H22A	120.1	F3C—B1—F3B	48.0 (18)
C22—C23—C18	120.6 (3)	F4A—B1—F3B	137.4 (11)
C22—C23—H23A	119.7	F4B—B1—F3B	101.5 (14)
C18—C23—H23A	119.7	F1—B1—F3B	100.6 (9)
C29—C24—C25	118.7 (2)	F2—B1—F3B	93.8 (7)
C29—C24—P1	119.1 (2)		
